# Hyperkinetic and Hypokinetic Movement Disorders in SSPE: A Systematic Review of Case Reports and Case Series

**DOI:** 10.5334/tohm.875

**Published:** 2024-05-14

**Authors:** Ravindra Kumar Garg, Shweta Pandey, Hardeep Singh Malhotra, Amita Jain, Ravi Uniyal, Neeraj Kumar, Imran Rizvi

**Affiliations:** 1 Department of Neurology, King George’s Medical University, Lucknow, India; 2 Department of Microbiology, King George’s Medical University, Lucknow, India

**Keywords:** Measles, Movement Disorders, Dystonia, Basal ganglion

## Abstract

**Background:**

Subacute Sclerosing Panencephalitis (SSPE) typically presents with periodic myoclonus; however, a spectrum of movement disorders including dystonia, chorea, tremor, and parkinsonism have also been described. This review aims to evaluate the array of movement disorders in SSPE, correlating them with neuroimaging findings, disease stages, and patient outcomes.

**Methods:**

A comprehensive review of published case reports and case series was conducted on patients with SSPE exhibiting movement disorders other than periodic myoclonus. PRISMA guidelines were followed, and the protocol was registered with PROSPERO (2023 CRD42023434650). A comprehensive search of multiple databases yielded 37 reports detailing 39 patients. Dyken’s criteria were used for SSPE diagnosis, and the International Movement Disorders Society definitions were applied to categorize movement disorders.

**Results:**

The majority of patients were male, with an average age of 13.8 years. Approximately, 80% lacked a reliable vaccination history, and 39% had prior measles infections. Dystonia was the most common movement disorder (49%), followed by parkinsonism and choreoathetosis. Rapid disease progression was noted in 64% of cases, with a disease duration of ≤6 months in 72%. Neuroimaging showed T2/FLAIR MR hyperintensities, primarily periventricular, with 26% affecting the basal ganglia/thalamus. Brain biopsies revealed inflammatory and neurodegenerative changes. Over half of the patients (56%) reached an akinetic mute state or died.

**Conclusion:**

SSPE is associated with diverse movement disorders, predominantly hyperkinetic. The prevalence of dystonia suggests basal ganglia dysfunction.

## Introduction

Subacute sclerosing panencephalitis (SSPE) is a progressive and fatal neurological disorder resulting from persistent brain infection with a mutated measles virus. SSPE manifests with cognitive deterioration, periodic myoclonus, and later akinetic rigid state. SSPE typically affects children of 5 to 15 years of age. SSPE leads to death within 1–3 years from onset. In its acute-fulminant form, it can result in death or an akinetic rigid state within 6 months. Periodic EEG patterns and characteristic neuroimaging help in making the diagnosis of SSPE. Diagnostics involve detecting elevated measles antibody levels in cerebrospinal fluid (CSF) [1,2,3].

SSPE is characterized by characteristic periodic myoclonus. In addition to periodic myoclonus, other movement disorders like dystonia, chorea, tremor, and parkinsonism have also been described as isolated case reports. Our review aims to explore the spectrum of other movement disorders in SSPE and their relationship with imaging findings, disease stage, and outcomes.

## Methods

Our comprehensive review examined published case studies and series involving patients with SSPE, focusing on those who exhibited a movement disorder other than periodic myoclonus. We adhered to the Preferred Reporting Items for Systematic Reviews and Meta-Analyses (PRISMA) guidelines throughout our systematic review. The study protocol was registered with PROSPERO (PROSPERO 2023 CRD42023434650) [4].

## Search strategy

We searched PubMed, Scopus, Embase, and Google Scholar databases. For the Google Scholar database, we limited our search to the initial 50 pages. We did not apply any language constraints while searching these databases. For articles not originally in English, we utilized “Google Translate” to render them in English. The search string we used was “((((((((Movement disorder) OR (Chorea)) OR (Dystonia)) OR (Tremors)) OR (Parkinsonism)) OR (Athetosis)) OR (repetitive behavior)) OR (Choreo-athetosis)) AND (Subacute sclerosing panencephalitis)”. The last search was done on 25 November 2023. All case reports, case series, or cohort studies, if individual patient data were available, were included in the review.

## Eligibility criteria

In this review, we focused on cases diagnosed with SSPE, who exhibited movement disorders other than periodic myoclonus. Diagnosis of SSPE based on Dyken’s criteria [5]. The definition of various movement disorders, we adhered to the definitions given by the International Movement Disorders Society [6] (Supplementary Table-1).

## Study selection

Two reviewers independently (SP, RKG) selected the studies based on the above-mentioned criteria, and any disagreement was resolved by mutual discussion. The selection of studies was performed in two steps. In the first step, the two reviewers independently screened titles and abstracts. In the second step, full articles of the selected studies were obtained. Any subsequent exclusion was recorded with a relevant reason. Duplicate records were managed using the EndNote 21web tool. Two reviewers (IR and RKG) independently conducted the duplicate removal process. Any issues that arose were resolved with the assistance of another reviewer (HSM).

## Data extraction

Data extraction was independently performed by two reviewers (NK and RU), and any disagreements were resolved through mutual discussion. Data was recorded on a Microsoft Excel sheet. A predetermined data extraction sheet was utilized for this purpose. This task was carried out by four reviewers (SP, RK, HSM, and NK). In the event of a dispute, assistance was sought from a fifth reviewer (AJ).

## Risk of bias (quality) assessment

The evaluation of the case reports’ quality was conducted across four domains: selection, ascertainment, causality, and reporting. A case report that satisfies the criteria in all domains is classified as “good quality.” Should it comply with three out of the four domains, it is considered “fair quality.” However, if it meets the criteria in only one or two of the domains, then it is described as “poor quality [7,8].”

## Data analysis

Data analysis was conducted using Microsoft Excel. We extracted the required data and consolidated it into an Excel file. The gathered information encompassed a variety of details: the primary author, the report’s country of origin, patient demographics, the status of early measles vaccinations, instances of childhood measles infections, the duration of the illness, various movement disorders, additional clinical findings, details of the diagnostic evaluations performed, neuroimaging data, and patient outcomes. Our analysis concentrated on the descriptive and qualitative dimensions of the data. For categorical variables, we used frequencies and percentages, while for continuous variables, we reported using means, medians, or ranges.

## Results

**Figure 1:**
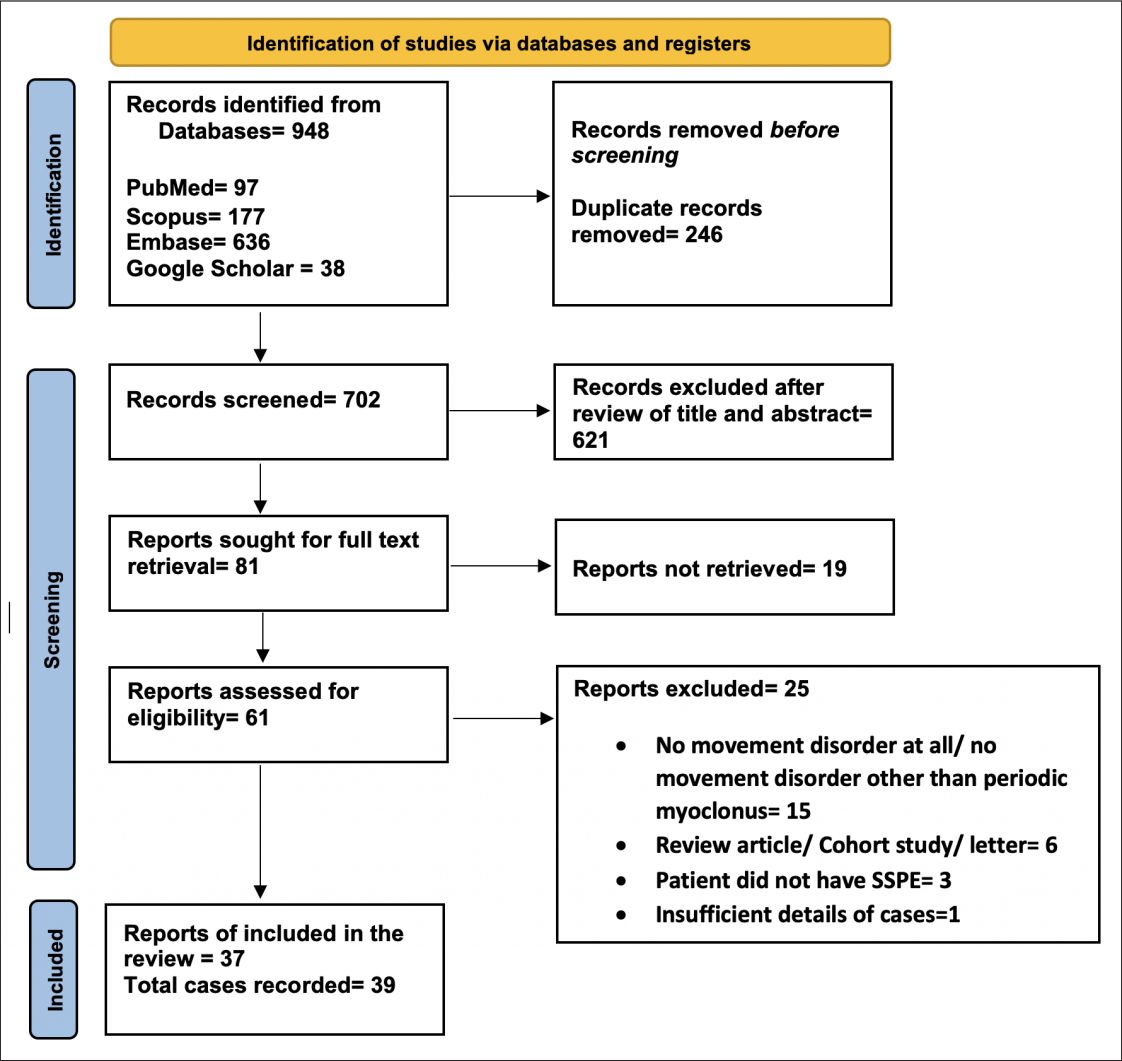
PRISMA flow diagram of the study depicts the procedure of selecting articles for the systematic review.

Our search resulted in 37 reports with details of 39 patients (Supplementary Table-2). We compiled our data as per PRISMA guidelines (Supplementary Table-3 – PRISMA checklist). Figure 1 shows the PRISMA flowchart for our systematic review. Table 1 presents a synopsis of the demographic, clinical, neuroimaging, and brain biopsy data for SSPE cases exhibiting a variety of movement disorders.

**Table 1:** Summary of epidemiological, clinical features, neuroimaging findings, histopathological features and outcome of patients of SSPE with various movement disorder (n = 39).


Age (in years)	Mean = 13.8

	Median = 14

	Range = 4–63

	Interquartile Range = 10

Sex	Female = 13 (33.3%)

	Male = 26 (66.7%)

Measles vaccination	Yes = 8 (20.5%)

	No/NA = 31(79.5%)

Childhood measles	Yes = 15 (38.5%)

	No or NA = 24 (61.5%)

Geographical areas of reported cases (total 37 reports)	India = 23 (62.2%)

	Other country in Asia = 1 (2.7%)

	Europe, except Turkey = 3 (8.1%)

	Turkey = 5 (13.5%)

	American continent = 4 (10.8%)

	Australia = 1 (2.7%)

Duration of illness	Sudden = 2 (5.2%)

	Within a week = 3 (7.8%)

	>1week up to 1 month = 8 (20.5%)

	>1 month to 6 months = 15 (38.5%)

	>6 months = 9 (23.7%)

	NA = 2 (5.2%)

Course of the disease	Acute fulminant = 25 (64%)

	Chronic = 11 (28.2%)

	NA = 3 (7.8%)

Movement disorder other than periodic myoclonus	Dystonia = 19 (48.7%)

	PISA syndrome = 3 (7.8%)

	Generalised = 11 (28.2%)

	Focal = 3 (7.8%)

	Hemidystonia = 2 (5.2%)

	Parkinsonism/hemi-parkinsonism = 8 (20.5%)

	Choreo-athetosis = 5 (12.8%)

	Chorea = 2 (5.2%)

	Tics = 2 (5.2%)

	Focal myoclonus = 1 (2.6%)

	Tremors and hyperkinesia = 1(2.6%)

	Neuroleptic malignant syndrome = 1(2.6%)

Neuroimaging	NA = 1(2.6%)

	Normal = 10 (25.6%)

	T2/FLAIR hyperintensity = 28 (71.8%)

	Basal ganglion = 8 (20.5%)

	Brain stem = 3(7.8%)

	Corpus callosum = 2 (5.2%)

	Thalamus = 2 (5.2%)

	Periventricular = 21 (53.8%)

	Whole frontal lobe =1 (2.6%)

	Cystic lesion = 1 (2.6%)

Brain biopsy	NA = 36 (92.3%)

	Done = 3 (7.8%)

Outcome	Deteriorated/ died = 22 (56.4%)

	Survived/ Improved = 8 (20.5%)

	NA = 9 (23.7%)


NA = not available; FLAIR = Fluid attenuated inversion recovery.

The supplemental Word file contains the critical appraisal reports for each included case, following the evaluation system outlined by Murad and colleagues. All the 39 cases being reviewed were categorized as good or fair (Supplementary Table-4).

The mean age of SSPE patients was 13.8 years (median 14 years, range 4–63 years), with a male predominance (21:11). A significant portion (80%) of patients lacked documentation of childhood measles vaccination or had an unreliable vaccination history. In 39% of cases, a history of childhood measles infection was noted. The majority (73.3%) of reports concerning SSPE patients presenting with diverse movement disorders originated from India and Turkey (Table 1).

Dystonia, parkinsonism, and choreoathetosis were the three most common movement disorders (82%) in SSPE cases. Dystonia was recorded in approximately 49% of SSPE patients. Chorea, tics, focal myoclonus, tremors, and neuroleptic malignant syndrome were other types of movement disorders that were described in SSPE patients. In approximately 64% (25/39) cases the course of SSPE was rapidly progressive. In approximately 72% (28/39) duration of illness was illness was 6 months or less.

Neuroimaging abnormalities were detected in 74% (29/39) of patients. T2/FLAIR MR hyperintensities were the most frequently noted (72%; 28/39) neuroimaging abnormality. Hyperintensities in 54% (21/39) of cases were predominantly located in periventricular regions. In 10 cases (26%) basal ganglion and/or thalamus was affected.

Brain biopsy findings were documented in three patients. Histopathological examination of brain tissue revealed inflammatory cell infiltration, elevated levels of activated microglial cells, diffuse or focal demyelination, the presence of inclusion bodies in neurons and oligodendroglia, as well as indications of neuronal loss.

Among 39 patients, 22 (56%) deteriorated to the akinetic mute stage or died during the follow-up period. For the rest 17 patients either patients, authors reported improvement following treatment, or information regarding outcome was not available.

## Discussion

We compiled 37 reports describing 39 patients. Dystonia, parkinsonism, and choreoathetosis were the three most common movement disorders, that were described in SSPE patients. Half of these cases were dystonia. The majority of these patients had acute-fulminant form of SSPE. In approximately 25% of cases, basal ganglionic/ thalamic neuroimaging abnormalities were noted.

In the cohort study, Garg et al reported that in 50 children with SSPE, over half of the children (56%) presented with movement disorders, predominantly myoclonus (92%). Other observed disorders included ataxia (18%), chorea-athetosis (14%), dystonia (12%), tremors (8%), repetitive behavior (8%), and parkinsonism (6%). Movement disorders were the presenting features in 7 cases [9].

Repetitive behaviors are defined as stereotypic body movements to impulsive/compulsive behaviors. In a case series of six patients with SSPE, a diverse array of repetitive behaviors was observed. All patients exhibited upper limb repetitive movements such as clapping, finger-clicking, hand rubbing, flailing, and dystonic posturing. Additionally, three patients showed lower limb movements, including heel rubbing, pelvic thrusts with leg flexion-extension, and foot tapping. Vocal repetitive behaviors were also noted, including palilalia, whistling, grunting coupled with spitting, and pathological crying. Notably, in two of the patients, these repetitive behaviors were the initial symptoms. There are certain differences between tics and repetitive behaviors. Repetitive Behaviors are repeated actions that may appear purposeful but are excessive. They can range from simple movements like hand-flapping to complex sequences of actions. Tics are sudden, rapid, recurrent, nonrhythmic motor movements or vocalizations. They are often experienced as an irresistible urge that can be temporarily suppressed [10].

The pathogenesis of movement disorders involves complex interactions within the brain, particularly focusing on the basal ganglia and the thalamus. In many patients of SSPE basal ganglion are dominantly affected [11]. The basal ganglia are a group of nuclei that process information related to movements and are connected with the cerebral cortex, thalamus, and other brain areas. Their primary function is to regulate voluntary motor control, procedural learning, and other cognitive and emotional activities. Movement disorders arise when there is a dysfunction in these regions. Hypokinetic disorders, such as Parkinson’s disease, occur due to an overactive inhibitory output from the basal ganglia to the thalamus, leading to decreased mobility or stiffness. Hyperkinetic disorders, like hemiballismus, are the result of too little inhibitory output, causing excessive and involuntary movements. The exact causes of movement disorders are not fully understood but are thought to involve imbalances in inhibitory and excitatory circuits influenced by the stroke’s vascular damage. The neurotransmitters GABA and dopamine, which are crucial in motor control, are particularly implicated in these disorders. Recent research has highlighted the role of oscillatory activities in the basal ganglia, thalamus, and cortex. In conditions like Parkinson’s disease, abnormal rhythms, particularly in the β-band frequency, disrupt the basal ganglia-thalamocortical circuits, leading to motor symptoms. Conversely, higher frequency oscillations may be associated with normal functions [12,13,14].

There are certain limitations to our review. First, the reliance on case reports and series may introduce selection bias, as these sources often highlight atypical or severe presentations, which may not represent the broader population of SSPE patients. The quality assessment of case reports across four domains provides a measure of rigor; however, the subjective nature of these assessments and the potential for inter-reviewer variability can affect consistency.

In conclusion, SSPE is associated with a range of hyperkinetic and hypokinetic movement disorders, with generalized dystonia being the most common. The primary cause of movement disorders in SSPE appears to be dysfunction of the basal ganglia.

## Additional File

The additional file for this article can be found as follows:

10.5334/tohm.875.s1 Supplementary File. Supplementary Tables 1 to 4.
