# Supplementary material for: Hyperkinetic and Hypokinetic Movement Disorders in SSPE: A Systematic Review of Case Reports and Case Series
Source: Tremor Other Hyperkinet Mov (N Y). 2024 May 14;14:27. doi: 10.5334/tohm.875 (PMC11100530; doi:10.5334/tohm.875)
Supplement: Supplementary File. — Supplementary Tables 1 to 4. [file tohm-14-1-875-s1.zip › tohm-875_garg_s1/Supplementary Table-1.docx]

Supplementary Table-1: Definitions of various movement disorders as per described by International Parkinson and Movement Disorder Society*.

| **Terms** | **Definitions** |
| --- | --- |
| **Chorea** | Chorea is defined as brief, abrupt, irregular, unpredictable, non-stereotyped movements. |
| **Dystonia** | Dystonia is defined as sustained or intermittent muscle contractions causing abnormal postures. Hemidystonia is a type of dystonia that affects one side of the body, including the face, arm, and leg. |
| **Pisa syndrome** | Pisa syndrome is defined as a reversible lateral bending of the trunk with a tendency to lean to one side. |
| **Dystonic storm or status dystonicus** | Status dystonicus is a characterised by a sudden, severe exacerbation of symptoms with widespread and intense muscle contractions. |
| **Tremor** | Tremor is an involuntary, rhythmic, oscillatory movement of a body part. |
| **Tics** | Tics are sudden, irregular movements or sounds resembling voluntary actions, often intense, repetitive, socially inappropriate, usually preceded by a "premonitory urge," and can be briefly suppressed. |
| **Myoclonus** | Myoclonus is defined as sudden, brief, involuntary jerks of a muscle or group of muscles. |
| **Athetosis** | Athetosis is a movement disorder marked by slow, continuous, twisting movements, especially of the hands. |
| **Dyskinesias** | Dyskinesias are uncontrolled, involuntary movements affecting the face, arms, legs, or trunk, manifesting as anything from tremors and tics to more extensive full-body motion. |
| **Parkinsonism** | Parkinsonism is characterised by bradykinesia, or slowness with decrement and degradation of repetitive movements. |

*International Parkinson and Movement Disorder Society. About Movement Disorders. <https://www.movementdisorders.org/MDS/About/Movement-Disorder-Overviews.htm> Assessed on 11 December, 2023.
