# Supplementary material for: Hyperkinetic and Hypokinetic Movement Disorders in SSPE: A Systematic Review of Case Reports and Case Series
Source: Tremor Other Hyperkinet Mov (N Y). 2024 May 14;14:27. doi: 10.5334/tohm.875 (PMC11100530; doi:10.5334/tohm.875)
Supplement: Supplementary File. — Supplementary Tables 1 to 4. [file tohm-14-1-875-s1.zip › tohm-875_garg_s1/Supplementary Table-1.pdf]

Supplementary Table-1: Definitions of various movement disorders as per described by International Parkinson and Movement Disorder Society*.

**Terms**	**Definitions**
**Chorea**	Chorea is defined as brief, abrupt, irregular, unpredictable, non-stereotyped movements.
**Dystonia**	Dystonia is defined as sustained or intermittent muscle contractions causing abnormal postures. Hemidystonia is a type of dystonia that affects one side of the body, including the face, arm, and leg.
**Pisa syndrome**	Pisa syndrome is defined as a reversible lateral bending of the trunk with a tendency to lean to one side.
**Dystonic storm or status dystonicus**	Status dystonicus is a characterised by a sudden, severe exacerbation of symptoms with widespread and intense muscle contractions.
**Tremor**	Tremor is an involuntary, rhythmic, oscillatory movement of a body part.
**Tics**	Tics are sudden, irregular movements or sounds resembling voluntary actions, often intense, repetitive, socially inappropriate, usually preceded by a "premonitory urge," and can be briefly suppressed.
**Myoclonus**	Myoclonus is defined as sudden, brief, involuntary jerks of a muscle or group of muscles.
**Athetosis**	Athetosis is a movement disorder marked by slow, continuous, twisting movements, especially of the hands.
**Dyskinesias**	Dyskinesias are uncontrolled, involuntary movements affecting the face, arms, legs, or trunk, manifesting as anything from tremors and tics to more extensive full-body motion.
**Parkinsonism**	Parkinsonism is characterised by bradykinesia, or slowness with decrement and degradation of repetitive movements.

*International Parkinson and Movement Disorder Society. About Movement Disorders. https://www.movementdisorders.org/MDS/About/Movement-Disorder-Overviews.htm Assessed on 11 December, 2023.
