# Supplementary material for: Hyperkinetic and Hypokinetic Movement Disorders in SSPE: A Systematic Review of Case Reports and Case Series
Source: Tremor Other Hyperkinet Mov (N Y). 2024 May 14;14:27. doi: 10.5334/tohm.875 (PMC11100530; doi:10.5334/tohm.875)
Supplement: Supplementary File. — Supplementary Tables 1 to 4. [file tohm-14-1-875-s1.zip › tohm-875_garg_s1/Supplementary Table-2.docx]

**Supplementary Table-2: Summary of epidemiological, clinical features, neuroimaging findings, histopathological features and outcome of patients with measles inclusion body encephalitis**

| Reference | Country | Age/sex | Measles vaccination / childhood measles | Duration of illness | Clinical features | Description of movement disorder | Type of movement disorder | Neuroimaging | CSF | Brain biopsy | Course | Treatment | Outcome |
| --- | --- | --- | --- | --- | --- | --- | --- | --- | --- | --- | --- | --- | --- |
| Youron et al 2023 | India | 13/M | Vaccinated  Had measles at 4 years | 1 month | fever and headache  Mental changes | Dystonic posturing of the left  upper and lower limbs and opisthotonus with grunting sounds  Periodic myoclonus  Coma | Choreo-athetosis | T2/FLAIR hyperintensity in both fronto-parietal regions | Lymphocytic pleocytosis with elevated protein  Antimeasles antibodies elevated | NA | Acute fulminant | Corticosteroids  Antiviral and anti-epileptic drugs | NA |
| Mondal et al 2023 | India | 63/F | NA | 3 months | Generalized abnormal movements, incontinence,  and sleep disturbances  Generalised myoclonus  Progressive encephalopathy | Involuntary, purposeless, asymmetric  flowing movements from one muscle group  Tongue and lip-smacking | Choreo-athetosis | T2/FLAIR hyperintensity in caudate and putamen | Antimeasles antibodies elevated | NA | Acute fulminant | Anti-epileptic drugs | Akinetic mute |
| Kaur et al 2023 | India | 23/M | NA | 1 month | Mental decline  Generalised myoclonus | Aright-sided tilt of the body in with the tendency to fall to the right side | PISA syndrome | T2/FLAIR hyperintensity in periventricular and deep white matter | Mildly elevated protein  Antimeasles antibodies elevated | NA | NA | Anti-epileptic drugs | Mild improvement |
| Harikrishna et al 2023 | India | 6/M | Not-vaccinated  Had measles at 3 years | 4 days | Fever and subsequently progressive encephalopathy  Myoclonus | Abnormal posturing of the left upper limb and recurrent falls while walking. | Status Dystonicus | T2/FLAIR hyperintensity in frontal, left parietal, and white matter, extending to corpus collosum | Antimeasles antibodies elevated | NA | Acute fulminant | Anti-epileptics and anti-psychotics | Akinetic mute |
| Garg et al 2023 | India | 9/M | NA | 5 months | Mental decline  Generalised myoclonus  Akinetic mute  Glasgow  Coma Scale score= 7 (E2V1M4) | Intermittent generalized axial  dystonic storm with flexion of upper limbs, an extension of  lower limbs, and opisthotonos | Generalized axial dystonic storm | T2/FLAIR hyperintensity in periventricular region  Multiple cystic  lesions present in periventricular white matter  Bilateral thalamic and basal ganglionic regions | Elevated protein  Antimeasles antibodies elevated | NA | Acute fulminant | Intrathecal  interferon-a  IVIG | Akinetic mute |
| Garg et al 2023 | India | 5/F | Vaccinated  Had measles at 6 months | 5 months | Recurrent falls  Seizures  Periodic myoclonus | Ballism-like limb movements  Short  bouts of laughter-like vocalization | Facial dystonia accompanying the dyskinetic limb movements | Normal | Normal  Antimeasles antibodies elevated | NA | Acute fulminant | Oral interferon-a  Antiepileptics | Mild improvement |
| Kalita et al 2022 | India | 20/F | Vaccinated | 9 months | A jerky neck movement  with speech arrest  Periodic myoclonus  Progressive encephalopathy  Patchy retinitis around the macula  Akinetic mute | Frequent  myoclonic jerks involving mainly axial and proximal  limbs, aggravated by loud sound | Focal myoclonus | T2/FLAIR hyperintensity in basal ganglion | Normal  Antimeasles antibodies elevated | NA | Acute fulminant | Antiepileptics | Akinetic mute |
| Holla et al 2022 | India | 26/M | Not-vaccinated | 3 years | Episodes of facio-brachial dystonic seizure  Mental decline | Episodes of jerks in left  upper limb, face, and  leg. | Faciobrachial dystonic seizure | Normal | Elevated protein  Antimeasles antibodies elevated | NA | Chronic | Intrathecal interferon  alfa-2b and an antiepileptic | Stable |
| Cornelius et al 2022 | India | 10/F | NA | 3 months | Difficulty in walking  Motor slowness  frequent backward falls while walking, slurring  of speech with reduced speech output  Progressive encephalopathy | Bradykinesia, rigidity, rest and postural  tremors, mask-like facies | Parkinsonism | T2/FLAIR hyperintensity in basal ganglion and subcortical frontal white matter | Antimeasles antibodies elevated | NA | Acute fulminant | Levodopa-carbidopa  Later antiepileptic drugs | Died |
| Regmi et al 2021 | India | 14/M | Non- vaccinated | 15 days | Seizures  Mental decline | Bradykinesia  Tremors  Masked facies | Parkinsonism | T2/FLAIR hyperintensity | Antimeasles antibodies elevated | NA | Acute fulminant | Intrathecal Interferon alpha | Akinetic mute |
| Uniyal et al 2021 | India | 17/M | Had measles at 1 year | 5 years | Seizures, abnormal postures of arms and legs  Decline in scholastic performance | Abnormal posturing of all four limbs  Limbs dystonia characterized by flexion at elbows, wrists, hips, and knees  Bruxism | Bruxism and generalized dystonia | Brain atrophy  Hot cross bun sign in mid pons  Molar tooth sign in ponto-mesencephalicjunction | Antimeasles antibodies elevated | NA | Chronic | Antiepileptics | Akinetic mute |
| Reddy et al 2021 | India | 18/F | Vaccinate | 1 month | Difficulty in walking  Slurring of speech and difficulty in swallowing  Cognitive decline  Myoclonus | Abnormal twisting movements of left  upper limb followed by lower limb progressed to  trunk and right side  Axial rigidity and bradykinesia | Generalised dystonia  Parkinsonism | Normal | Antimeasles antibodies elevated | NA | Acute fulminant | Intrathecal interferons, Isoprinosine  Trihexyphenidyl, and levodopa | NA |
| Khilari et al 2020 | India | 7/F | NA | Sudden | Status epilepticus  Generalised recurrent myoclonic jerks  Coma | Sustained posturing of both upper limbs with flexion of both wrists and elbows and lower limbs at knees | Generalised dystonia | T2/FLAIR hyperintensity in basal ganglion  Mild cortical ribboning on DWI | Normal  Antimeasles antibodies elevated | NA | Acute fulminant | Antiepileptics | Mild improvement |
| Guruswamy and Kurpad 2020 | India | 17/M | NA | 2 years | Seizures  Slow walking  Behaviour abnormality  Mental decline | Cogwheel rigidity, dystonia was pre­sent in all four lower limbs. | Parkinsonism | T2/FLAIR hyperintensity in temporal and occipital lobes bilaterally | Elevated protein  Antimeasles antibodies elevated | NA | Chronic | Antiepileptics  DOPA and carbidopa  Isoprinosine | NA |
| Tandra et al 2019 | India | 10/M | Had measles at 4 years | 2 months | Mental decline  Frequent fall  Abnormal behavior  Periodic myoclonus | Abnormal fidgety limb movements  aggravated by action or excitement and disappeared in sleep.  Flinging movements  flowing movements from one muscle  group to another.  Movements  involved both distal and proximal muscles.  Orolingual dyskinesia | Choreoathetosis | T2/FLAIR hyperintensity in basal ganglion and subcortical temporal lobes | Elevated protein  Antimeasles antibodies elevated | NA | Acute fulminant | Antiepileptics  Isoprinosine | Stable |
| Pandey et al 2018 | India | 8/M | Had measles at 3 years | 3 months | Mental decline  Myoclonus | Lateral bending of the trunk toward the right-side during standing  or walking | Pisa syndrome | T2/FLAIR hyperintensity in temporal and parietal lobes bilaterally | Normal  Antimeasles antibodies elevated | NA | Acute fulminant | Antiepileptics  Trihexyphenidyl  levodopa plus carbidopa | No response |
| Goswami and Roy 2018 | India | 8/F | Not-vaccinated | 2 weeks | Severe encephalopathy | Dystonic posturing right side  Hemi-dystonia involving right-sided limbs | Dystonic Storm | T2/FLAIR hyperintensity in occipital lobes and basal ganglion | Antimeasles antibodies elevated | NA | Acute fulminant | Antiepileptics  Trihexyphenidyl  levodopa plus carbidopa | Partial response |
| Garg et al 2018 | India | 32/M | NA | 3 weeks | Fever  Headache  Vision loss  Gait ataxia  Mental decline  Myoclonus | Stiffness of all four limbs  Severe rigidity of all four limbs | Neuroleptic malignant syndrome | T2/FLAIR hyperintensity in parieto- occipital region | Normal  Antimeasles antibodies elevated | NA | Acute fulminant | Methylprednisolone  Interferon alfa-  and isoprinosine  Antiepileptics | Severe septic shock and died |
| Singhi et al 2015 | India | 4/F | Had fever with rash | 2 months | Vision loss  Mental decline  Severe encephalopathy  Chorioretinitis | Twisting movements of the limbs  Increased on agitation and decreased on sleep | Choreoathetosis | T2/FLAIR hyperintensity in the midbrain,  ventral pons, and splenium of the corpus callosum | Normal  Antimeasles antibodies elevated | NA | Acute fulminant | Oral isoprinosine | Not improved  Akinetic mute |
| Raina et al 2015 | Argentina | 16/F | Vaccinated | 3 months | Progressive mental decline  Dystonic and paroxysmal movements | Dystonic  movements in her upper right limb  progressed over 3 months  involving her four limbs with retrocollis and jaw-opening dystonia | Dystonic paroxysmal episodes like kinesigenic paroxysmal dyskinesias | T2/FLAIR hyperintensity in periventricular region and basal ganglion | Antimeasles antibodies elevated | NA | Acute fulminant | Antiepileptics  intraventricular (IV) Interferon-a2B therapy | Akinetic mute |
| Malhotra and Garg 2015 | India | 25/M | Had measles at 3 years | 3 months | Recurrent falls  Mental decline  Myoclonus | Slowness of movements  Body tilt | Pisa syndrome and striatal toe | T2/FLAIR hyperintensity in periventricular regions | Cells were elevated  Antimeasles antibodies elevated | NA | Acute fulminant | Clonazepam and  Trihexyphenidyl  Interferon-alpha | Died |
| Kannan et al 2015 | India | 8/M | NA | 1 month | Mental decline  Myoclonus | Abnormal twisting of lips and tongue with slurring of speech, drooling, and difficulty in swallowing  Bradykinesia, generalized dystonia, orofacial-dyskinesia, rigidity in all four limbs | Dystonia-Parkinsonism | Normal | Antimeasles antibodies elevated | NA | Acute fulminant | Antiepileptics | Further deterioration |
| Bozlu et al 2015 | Turkey | 12/M | Vaccinated | 2 months | Periodic myoclonus | Difficulty  in walking  Dystonia, left-sided  Tremors  Generalized stiffness | Juvenile Parkinson disease | T2/FLAIR hyperintensity in periventricular parietal regions | Antimeasles antibodies elevated | NA | Acute fulminant | Levodopa, trihexyphenidyl, tetrabenazine  and clonazepam  IVIG | Improved |
| Serin et al 2014 | Turkey | 11/M | NA | 1 week | Speech impairment and gait instability | Shaking in the right hand and limping in the  right leg | Hemidystonia | T2/FLAIR hyperintensity in  the basal ganglia and parieto-occipital region | Antimeasles antibodies elevated | NA | Chronic | Antiepileptics | Gradual deterioration |
| Roceanu et al 2013 | Romania | 19/M | Measles at 7 months | NA | Mental decline  Vision loss | Involuntary movements of the left upper limb  Unilateral myoclonic jerks  choreoathetosis of the left  fingers | Choreoathetosis and unilateral myoclonus | Normal | Antimeasles antibodies elevated | NA | NA | NA | NA |
| Dey and Bhattacharya 2013 | India | 10/M | Had measles at 2 years | 5 months | Ataxia  Periodic myoclonus | Complex tics involving  shoulder and facial muscles  Stereotypic repetitive movements and bradykinesia | Tics | T2/FLAIR hyperintensity in  parieto-occipital region | Antimeasles antibodies elevated | NA | Chronic | Isoprinosine  Antiepileptics | NA |
| Yiş 2012 | Turkey | 14/M | NA | 2 years | Drop attacks and behavioral changes  Rapidly progressive dystonia, hyperpyrexia | Paroxysmal dystonic attacks | Status Dystonicus  Myoglobinuria | NA | NA | NA | NA | Isoprinosine  Carbamazepine  Baclofen | NA |
| Almeida et al 2012 | Brazil | 15/M | vaccinated | 3 years | Seizures  Myoclonus  Mental decline | Spontaneous laughter  Tongue tremor  Dystonia in upper and  lower limbs | Generalised dystonia | T2/FLAIR hyperintensity in both basal ganglion | Elevated protein  Antimeasles antibodies elevated | Brain autopsy=perivascular inflammatory cuffing, astro-microgliosis, neurono­phagia and Cowdry type A eosinophilic intranuclear inclusion bodies  Immunohistochemistry= measles | Chronic | Symptomatic | Died |
| Teber et al 2011 | Turkey | 11/M | Vaccinated  Had measles at 3 years | 1 week | Involuntary movement and gait disturbance  Complex partial seizure with secondary  Generalization  One month later progressive diffuse encephalopathy | Chorea  on upper extremities and on his tongue | Chorea | Normal | Antimeasles antibodies elevated | NA | Acute fulminant | Haloperidol  Isoprinosine  Carbamazepine  Clonazepam | Died |
| Fabian et al 2009 | Australia | 22/F | NA | 6 months | Vision loss  Tremors  Mental decline | Choreiform movements of the left upper  Limb  dystonia of the left leg | Chorea and focal dystonia | T2/FLAIR hyperintensity in occipital lobes | Normal  Antimeasles antibodies elevated | Brain autopsy= cellular infiltration astrogliosis, cortical neuronal loss, microglial nodules and perivascular  cuffing by lymphocytes  Neuronal intranuclear  eosinophilic inclusions were seen only within the hippocampus  Electron microscopy= viral nucleocapsids of a paramyxovirus. | Acute fulminant | Supportive | Died |
| Misra et al 2008 | India | 13/M | NA | 2 months | Difficulty in walking  Speech abnormality  Bradykinesia | Mask-like facies,  Tremor,  cogwheel rigidity, bradykinesia, and postural  instability | Parkinsonism | Normal | Antimeasles antibodies elevated | NA | Acute fulminant | Levodopa and carbidopa | NA |
|  |  | 15/M | Vaccinated | 3 months | Recurrent falls  Difficulty in walking  Speech abnormality  Mental decline  Periodic myoclonus | Masked facies  Hypophonic speech  Cogwheel rigidity, bradykinesia and impaired  postural reflexes | Parkinsonism | Normal | Antimeasles antibodies elevated | NA | Acute fulminant | NA | NA |
| Ondo and Verma 2002 | USA | 26/F | NA | 4 months | Vision loss  Recurrent falls  Progressive mental decline | Intermittent neck turning  facial  grimacing and then arm and leg extension and twisting  Dystonic extension and rotation of the  arms, extension of the right leg, rotation of the neck, and  contraction of face | Generalised dystonia | Normal | Antimeasles antibodies elevated | NA | Acute fulminant | Antiepileptics  Antiviral medication, amantadine, and  prednisone | Died |
| Scheidt et al 2001 | Germany | 15/M | NA | 1 year | Gait unsteadiness  Falls  Speech difficulties  Progressive mental decline  Myoclonus | Dystonia in his left hand | Focal dystonia | T2/FLAIR hyperintensity in the thalamic and hypothalamic regions  Later in brain stem and periventricular regions | Normal  Antimeasles antibodies elevated | NA | Chronic | Isoprinosine | Akinetic mute |
| Dimova and Bojinova 2000  A report of 3 patientS, 2 had movement disorders. | Turkey | 11/F | Had measles at 2 years  Unvaccinated | Sudden | Progressive encephalopathy  Seizures  Periodic myoclonus | Action tremor in the right hand | Tremors and hyperkinesia | T2/FLAIR hyperintensity in whole of right frontal lobe | Antimeasles antibodies elevated | NA | Acute fulminant | Isoprinosine | Akinetic mute |
|  |  | 6/F | Had measles at 6 months  Unvaccinated | NA | Progressive encephalopathy  Periodic myoclonus | Left-leg dystonia and hemi parkinsonism. | Hemidystonia- hemi parkinsonism | T2/FLAIR hyperintensity in  parieto-occipital region | Antimeasles antibodies elevated | NA | Chronic | Isoprinosine | NA |
| Vela et al 1997 | Spain | 30/M | Had measles at 8 months  Unvaccinated | 3 years | Movements of neck that were considered as “tics”.  Periodic myoclonus  Encephalopathy | Movements of neck that were considered as “tics”. | Tics | T2/FLAIR hyperintensity in  parieto-occipital region | Antimeasles antibodies elevated | NA | Chronic | Carbamazepine  Isoprinosine | Akinetic mute |
| Doh et al 1997 | Korea | 26/M | Had measles at 13 years | 1 month | Bradykinesia  Slurred speech  Gait abnormality  Myoclonic jerks | Masked facies  Bradykinesia  Rigidity  Parkinsonian gait | Parkinsonism | Normal later brain atrophy | Antimeasles antibodies elevated | Brain biopsy- glial and inflammatory changes  Neuronal loss  Perivascular cuffing  Demyelination  Inclusions were not seen. | Chronic | Interferon  Amantadine  Isoprinosine  Rifampicin | Died |
| Jankovic 1988 | USA | 16/M  Delayed milestones | NA | 2 years | Left sided weakness  Gait abnormality  Chorioretinal scars | Bradykinesia  Cog wheel Rigidity  Tremors  Masked facies  Sialorrhea  Stooped posture  Retropulsion | Parkinsonism | T2/FLAIR hyperintensity in  parieto-occipital region | Antimeasles antibodies elevated | Brain biopsy- glial and inflammatory changes  Neuronal loss  Perivascular cuffing  Demyelination  Inclusion bodies were seen.  Electron microscopy= viral nucleocapsids of a paramyxovirus | Chronic | Carbidopa-levodopa  Amantadine | Stabilised |

**References**

Youron P, Mahajan S, Balaini N, Mehta S, Lal V. Fulminant SSPE Presenting as a Hyperkinetic Movement Disorder. Mov Disord Clin Pract. 2023;10(5):830-832. doi: 10.1002/mdc3.13686.

Mondal R, Deb S, Mahata M, Saha S, Lahiri D, Benito-León J. Subacute Sclerosing Panencephalitis in a 63-Year-Old Woman Presenting as Generalized Choreoathetosis. Neurohospitalist. 2023 Oct;13(4):381-393. doi: 10.1177/19418744231177105.

Kaur S, Singh AS, Prabhakar S, Singhvi JP, Mann HS, Kaul A. Pisa Syndrome in Subacute Sclerosing Panencephalitis: A Case Report and Review of the Literature. J Mov Disord. 2023 Sep;16(3):336-338. doi: 10.14802/jmd.23052.

Harikrishna GV, Chowdary MR, Vengalil S, Nalini A, Yadav R. Status Dystonicus in Subacute Sclerosing Panencephalitis-A Rare Presentation in Emergency. Neurol India. 2023 Sep-Oct;71(5):994-997. doi: 10.4103/0028-3886.388104.

Garg RK, Pandey S, Nigam H, Keerthiraj DB, Rizvi I, Kumar N, Uniyal R, Malhotra HS, Sharma PK. Case Report: An Unusual Case of Subacute Sclerosing Panencephalitis with Distinctive Clinical and Neuroimaging Features. Am J Trop Med Hyg. 2023 Mar 13;108(5):1025-1027. doi: 10.4269/ajtmh.22-0731.

Garg D, Kakkar V, Sharma S. Periodic Laughter-like Episodes in Subacute Sclerosing Panencephalitis-Is it Gelastic Myoclonus? Mov Disord Clin Pract. 2023 Aug 7;10(10):1547-1548. doi: 10.1002/mdc3.13848.

Kalita J, Chaudhary SK, Kumar B, Jadhav M. Case Report: Focal Myoclonus with a Striatal Lesion as a Presentation of Subacute Sclerosing Panencephalitis. Am J Trop Med Hyg. 2022 May 9;106(6):1729–31. doi: 10.4269/ajtmh.22-0046.

Holla VV, Chaithra SP, Prasad S, Kamble N, Pal PK, Yadav R. Faciobrachial dystonic seizure-like events in a patient with subacute sclerosing panencephalitis. Annals of Movement Disorders. 2022 May 1;5(2):121-4. DOI: 10.4103/AOMD.AOMD_41_21

Cornelius LP, Elango N, Jeyaram VK. Akinetic rigid syndrome as a presenting feature of subacute sclerosing pan encephalitis. Neurology Asia. 2022 Mar 31;27(1):191-4.

Regmi J, Airani ZK, Mohan S, Patil S, Shetty V, Moulick N. Subacute Sclerosing Panencephalitis Presenting Like Parkinsonism: A Rare Case. International Journal of Science and Research. 2020; 10:1192-1193. DOI:10.21275/SR211225090518

Uniyal R, Garg RK, Malhotra HS, Tejan N, Kumar N, Pandey S, Shekhar R. A Case of Subacute Sclerosing Panencephalitis: Some Unusual Clinico-Radiological Manifestations. Neurol India. 2021 Sep-Oct;69(5):1446-1448. doi: 10.4103/0028-3886.329564.

Reddy RB, Joshi D, Kumar A. Subacute Sclerosing Panencephalitis with An Atypical Presentation. Ann Indian Acad Neurol. 2021 Nov-Dec;24(6):946-947. doi: 10.4103/aian.AIAN_791_20.

Khilari ML, Sharma PK. Clinical conundrum: status epilepticus culminating into acute dystonia myoclonus. BMJ Case Rep. 2020 Feb 28;13(2):e233397. doi: 10.1136/bcr-2019-233397.

Guruswamy A, Kurpad K P. Interesting MRI finding in SSPE – a case report. Polish Annals of Medicine. 2020;27(1):45-7. https://doi.org/10.29089/2019.19.00088

Tandra HV, Roy PS, Sharma R, Bhatia V, Saini AG. Subacute Sclerosing Panencephalitis Presenting as Choreoathetosis and Basal Ganglia Hyperintensities. Neurohospitalist. 2019 Jan;9(1):26-29. doi: 10.1177/1941874418776902.

Pandey S, Tomar LR, Tater P. Pisa Syndrome in a Child With Subacute Sclerosing Panencephalitis. JAMA Neurol. 2018 Feb 1;75(2):255-256. doi: 10.1001/jamaneurol.2017.4092.

Goswami JN, Roy S. Dystonic Storm: An Atypical Presentation of Subacute Sclerosing Panencephalitis. Indian Pediatr. 2018 May 15;55(5):441.

Garg D, Reddy V, Singh RK, Dash D, Bhatia R, Tripathi M. Neuroleptic malignant syndrome as a presenting feature of subacute sclerosing panencephalitis. J Neurovirol. 2018 Feb;24(1):128-131. doi: 10.1007/s13365-017-0602-4.

Singhi P, Saini AG, Sankhyan N, Gupta P, Vyas S. Blindness, dancing extremities, and corpus callosum and brain stem involvement: an unusual presentation of fulminant subacute sclerosing panencephalitis. J Child Neurol. 2015 Jan;30(1):87-90. doi: 10.1177/0883073813520498.

Raina GB, Folgar SS, Garrido JP, Calvo DS, Olivos NA, Morera N, Moreno M, Roca MU, Micheli F. Secondary kinesigenic paroxysmal dyskinesias: Report of two unusual cases responsive to carbamazepine. Basal Ganglia. 2015 Mar 1;5(1):7-9.

Malhotra HS, Garg RK. Pearls & Oy-sters: Pisa syndrome: an unusual feature of adult-onset fulminant SSPE. Neurology. 2015 Jan 20;84(3):e12-4. doi: 10.1212/WNL.0000000000001161.

Kannan L, Jain P, Sharma S, Gulati S. Subacute sclerosing panencephalitis masquerading as rapid-onset dystonia-Parkinsonism in a child. Neurol India. 2015 Jan-Feb;63(1):109-10. doi: 10.4103/0028-3886.152678.

Bozlu G, Cobanogullari Direk M, Okuyaz C. Subacute sclerosing panencephalitis with parkinsonian features in a child: A case report. Brain Dev. 2015 Oct;37(9):901-3. doi: 10.1016/j.braindev.2015.02.008.

Serin HM, Bilen S, Cansu A. Subacute Sclerosing Panencephalitis Presenting with Hemidystonia. Medical Bulletin of Haseki/Haseki Tip Bulteni. 2014 Jun 1;52(2). DOI: 10.4274/haseki.1451

Roceanu A, Antochi F, Bajenaru O. Atypical clinical presentation of subacute sclerosing panencephalitis (SSPE). Romanian Journal of Neurology/ Revista Romana de Neurologie 2013;12:142-147.

Dey PK, Bhattacharya T. Subacute sclerosing panencephalitis with tics as first symptom. Indian Pediatr. 2013 Nov 8;50(11):1067-8.

Yiş U. Status dystonicus and rhabdomyolysis in a patient with subacute sclerosing panencephalitis. Turk J Pediatr. 2012 Jan-Feb;54(1):90-1.

Almeida KJ, Brucki SMD, Duarte MIS, Pasqualucci CAG, Rosemberg S, Nitrini R. Basal ganglia lesions in subacute sclerosing panencephalitis. Dement Neuropsychol. 2012 Oct-Dec;6(4):286-289. doi: 10.1590/S1980-57642012DN06040014.

Teber S, Sezer T, Kafali M, Deda G. Subacute sclerosing panencephalitis with an atypical presentation: A case report. Journal of Pediatric Neurology 2011;9:127-130.

Fabian VA, Lee HY, Keith-Rokosh JL, de Souza JL, Stewart-Wynne E. A 22-year-old Australian woman with atypical subacute sclerosing panencephalitis diagnosed at postmortem. J Clin Neurosci 2010;17:1192-1194.

Misra AK, Roy A, Das SK. Parkinsonian presentation of SSPE: Report of two cases. Neurology Asia 2008;13:117-120.

Ondo WG, Verma A. Physiological assessment of paroxysmal dystonia secondary to subacute sclerosing panencephalitis. Mov Disord. 2002 Jan;17(1):154-7. doi: 10.1002/mds.10005.

Scheidt R, Schellenschmitt M, Dorstelmann D. Behavioural disturbances and dystonia as first manifestations of subacute sclerosing panencephalitis. AKTUELLE NEUROLOGIE. 2001 Mar 1;28(2):82-5.

Dimova P, Bojinova V. Subacute sclerosing panencephalitis with atypical onset: clinical, computed tomographic, and magnetic resonance imaging correlations. J Child Neurol. 2000 Apr;15(4):258-60. doi: 10.1177/088307380001500411.

Vela L, Garcia-Merino A, Escamilla C. Adult-onset subacute sclerosing panencephalitis first seen as craniocervical myoclonus. Mov Disord. 1997 May;12(3):462-4. doi: 10.1002/mds.870120335.

DOH W-B, KIM S-M, KIM S-Y, et al. Subacute sclerosing panencephalitis presenting as young adult onset parkinsonism. Journal of the Korean Neurological Association 1997:874-880.

Jankovic J, Armstrong D, Low NL, Goetz CG. Case 2, 1988. Congenital mental retardation and juvenile parkinsonism. Mov Disord. 1988;3(4):352-61. doi: 10.1002/mds.870030413.
