# Supplementary material for: Hyperkinetic and Hypokinetic Movement Disorders in SSPE: A Systematic Review of Case Reports and Case Series
Source: Tremor Other Hyperkinet Mov (N Y). 2024 May 14;14:27. doi: 10.5334/tohm.875 (PMC11100530; doi:10.5334/tohm.875)
Supplement: Supplementary File. — Supplementary Tables 1 to 4. [file tohm-14-1-875-s1.zip › tohm-875_garg_s1/Supplementary Table-2.pdf]

Supplementary Table-2: Summary of epidemiological, clinical features, neuroimaging findings, histopathological features and outcome of patients with measles inclusion body encephalitis

Reference	Country	Age/sex	Measles vaccination / childhood measles	Duration of illness	Clinical features	Description of movement disorder	Type of movement disorder	Neuroimaging	CSF	Brain biopsy	Course	Treatment	Outcome
Youron et al 2023	India	13/M	Vaccinated<br><br>Had measles at 4 years	1 month	fever and headache<br><br>Mental changes	Dystonic posturing of the left upper and lower limbs and opisthotonus with grunting sounds<br><br>Periodic myoclonus<br><br>Coma	Choreo-athetosis	T2/FLAIR hyperintensity in both fronto-parietal regions	Lymphocytic pleocytosis with elevated protein<br><br>Antimeasles antibodies elevated	NA	Acute fulminant	Corticosteroids<br><br>Antiviral and anti-epileptic drugs	NA
Mondal et al 2023	India	63/F	NA	3 months	Generalized abnormal movements, incontinence, and sleep disturbances<br><br>Generalised myoclonus<br><br>Progressive encephalopathy	Involuntary, purposeless, asymmetric flowing movements from one muscle group<br><br>Tongue and lip-smacking	Choreo-athetosis	T2/FLAIR hyperintensity in caudate and putamen	Antimeasles antibodies elevated	NA	Acute fulminant	Anti-epileptic drugs	Akinetic mute
Kaur et al 2023	India	23/M	NA	1 month	Mental decline<br><br>Generalised myoclonus	Aright-sided tilt of the body in with the tendency to fall to the right side	PISA syndrome	T2/FLAIR hyperintensity in periventricular and deep white matter	Mildly elevated protein<br><br>Antimeasles antibodies elevated	NA	NA	Anti-epileptic drugs	Mild improvement
Harikrishna et	India	6/M	Not-vaccinate	4 days	Fever and subsequently	Abnormal posturing of	Status Dystonicus	T2/FLAIR hyperinten	Antimeasles	NA	Acute fulminant	Anti-epilepti	Akinetic mute

al 2023			d<br><br>Had measles at 3 years		progressive encephalopathy<br><br>Myoclonus	the left upper limb and recurrent falls while walking.		sity in frontal, left parietal, and white matter, extending to corpus collosum	antibodies elevated			cs and anti-psychotics	
Garg et al 2023	India	9/M	NA	5 months	Mental decline<br><br>Generalised myoclonus<br><br>Akinetic mute<br><br>Glasgow Coma Scale score= 7 (E2V1M4)	Intermittent generalized axial dystonic storm with flexion of upper limbs, an extension of lower limbs, and opisthotonos	Generalized axial dystonic storm	T2/FLAIR hyperintensity in periventricular region<br><br>Multiple cystic lesions present in periventricular white matter<br><br>Bilateral thalamic and basal ganglionic regions	Elevated protein<br><br>Antimeasles antibodies elevated	NA	Acute fulminant	Intrathecal interferon-a<br><br>IVIG	Akinetic mute
Garg et al 2023	India	5/F	Vaccinated<br><br>Had measles at 6 months	5 months	Recurrent falls Seizures<br><br>Periodic myoclonus	Ballism-like limb movements<br><br>Short bouts of laughter-like vocalization	Facial dystonia accompanying the dyskinetic limb movements	Normal	Normal<br><br>Antimeasles antibodies elevated	NA	Acute fulminant	Oral interferon-a<br><br>Antiepileptics	Mild improvement
Kalita et al 2022	India	20/F	Vaccinated	9 months	A jerky neck movement with speech arrest<br><br>Periodic myoclonus<br><br>Progressive encephalopathy<br><br>Patchy	Frequent myoclonic jerks involving mainly axial and proximal limbs, aggravated by loud sound	Focal myoclonus	T2/FLAIR hyperintensity in basal ganglion	Normal<br><br>Antimeasles antibodies elevated	NA	Acute fulminant	Antiepileptics	Akinetic mute

					retinitis around the macula<br><br>Akinetic mute							
Holla et al 2022	India	26/M	Not-vaccinated	3 years	Episodes of facio-brachial dystonic seizure<br><br>Mental decline	Episodes of jerks in left upper limb, face, and leg.	Faciobrachial dystonic seizure	Normal	Elevated protein<br><br>Antimeasles antibodies elevated	NA	Chronic	Intrathecal interferon alfa-2b and an antiepileptic	Stable
Cornelius et al 2022	India	10/F	NA	3 months	Difficulty in walking<br><br>Motor slowness<br><br>frequent backward falls while walking, slurring of speech with reduced speech output<br><br>Progressive encephalopathy	Bradykinesia, rigidity, rest and postural tremors, mask-like facies	Parkinsonism	T2/FLAIR hyperintensity in basal ganglion and subcortical frontal white matter	Antimeasles antibodies elevated	NA	Acute fulminant	Levodopa-carbidopa Later antiepileptic drugs	Died
Regmi et al 2021	India	14/M	Non-vaccinated	15 days	Seizures Mental decline	Bradykinesia Tremors Masked facies	Parkinsonism	T2/FLAIR hyperintensity	Antimeasles antibodies elevated	NA	Acute fulminant	Intrathecal Interferon alpha	Akinetic mute
Uniyal et al 2021	India	17/M	Had measles at 1 year	5 years	Seizures, abnormal postures of arms and legs<br><br>Decline in	Abnormal posturing of all four limbs<br><br>Limbs dystonia	Bruxism and generalized dystonia	Brain atrophy<br><br>Hot cross bun sign in mid pons	Antimeasles antibodies elevated	NA	Chronic	Antiepileptics	Akinetic mute

					scholastic performance	characterized by flexion at elbows, wrists, hips, and knees<br><br>Bruxism		Molar tooth sign in ponto-mesencephalicjunction					
Reddy et al 2021	India	18/F	Vaccinate	1 month	Difficulty in walking<br>Slurring of speech and difficulty in swallowing<br>Cognitive decline<br><br>Myoclonus	Abnormal twisting movements of left upper limb followed by lower limb progressed to trunk and right side<br><br>Axial rigidity and bradykinesia	Generalised dystonia<br><br>Parkinsonism	Normal	Antimeasles antibodies elevated	NA	Acute fulminant	Intrathecal interferons, Isoprinosine<br><br>Trihexyphenidyl, and levodopa	NA
Khilari et al 2020	India	7/F	NA	Sudden	Status epilepticus<br><br>Generalised recurrent myoclonic jerks<br><br>Coma	Sustained posturing of both upper limbs with flexion of both wrists and elbows and lower limbs at knees	Generalised dystonia	T2/FLAIR hyperintensity in basal ganglion<br><br>Mild cortical ribboning on DWI	Normal<br><br>Antimeasles antibodies elevated	NA	Acute fulminant	Antiepileptics	Mild improvement
Guruswamy and Kurpad 2020	India	17/M	NA	2 years	Seizures<br>Slow walking<br>Behaviour abnormality<br><br>Mental decline	Cogwheel rigidity, dystonia was present in all four lower limbs.	Parkinsonism	T2/FLAIR hyperintensity in temporal and occipital lobes bilaterally	Elevated protein<br><br>Antimeasles antibodies elevated	NA	Chronic	Antiepileptics<br><br>DOPA and carbidopa<br><br>Isoprinosine	NA
Tandra et al 2019	India	10/M	Had measles at 4 years	2 months	Mental decline<br><br>Frequent fall	Abnormal fidgety limb movements aggravated	Choreoathetosis	T2/FLAIR hyperintensity in basal	Elevated protein<br><br>Antimea	NA	Acute fulminant	Antiepileptics<br><br>Isoprin	Stable

					Abnormal behavior<br><br>Periodic myoclonus	by action or excitement and disappeared in sleep.<br><br>Flinging movements flowing movements from one muscle group to another.<br><br>Movements involved both distal and proximal muscles.<br><br>Orolingual dyskinesia		ganglion and subcortical temporal lobes	sles antibodies elevated			osine	
Pandey et al 2018	India	8/M	Had measles at 3 years	3 months	Mental decline Myoclonus	Lateral bending of the trunk toward the right-side during standing or walking	Pisa syndrome	T2/FLAIR hyperintensity in temporal and parietal lobes bilaterally	Normal<br><br>Antimeasles antibodies elevated	NA	Acute fulminant	Antiepileptics<br><br>Trihexyphenidyl<br><br>levodopa plus carbidopa	No response
Goswami and Roy 2018	India	8/F	Not-vaccinated	2 weeks	Severe encephalopathy	Dystonic posturing right side Hemi-dystonia involving right-sided limbs	Dystonic Storm	T2/FLAIR hyperintensity in occipital lobes and basal ganglion	Antimeasles antibodies elevated	NA	Acute fulminant	Antiepileptics<br><br>Trihexyphenidyl<br><br>levodopa plus carbidopa	Partial response

Garg et al 2018	India	32/M	NA	3 weeks	Fever Headache Vision loss Gait ataxia Mental decline Myoclonus	Stiffness of all four limbs<br><br>Severe rigidity of all four limbs	Neuroleptic malignant syndrome	T2/FLAIR hyperintensity in parieto-occipital region	Normal<br><br>Antimeasles antibodies elevated	NA	Acute fulminant	Methylprednisolone<br><br>Interferon alfa- and isoprinosine<br><br>Antiepileptics	Severe septic shock and died
Singhi et al 2015	India	4/F	Had fever with rash	2 months	Vision loss Mental decline Severe encephalopathy<br><br>Chorioretinitis	Twisting movements of the limbs Increased on agitation and decreased on sleep	Choreoathetosis	T2/FLAIR hyperintensity in the midbrain, ventral pons, and splenium of the corpus callosum	Normal<br><br>Antimeasles antibodies elevated	NA	Acute fulminant	Oral isoprinosine	Not improved<br><br>Akinetic mute
Raina et al 2015	Argentina	16/F	Vaccinated	3 months	Progressive mental decline<br><br>Dystonic and paroxysmal movements	Dystonic movements in her upper right limb<br><br>progressed over 3 months involving her four limbs with retrocollis and jaw-opening dystonia	Dystonic paroxysmal episodes like kinesigenic paroxysmal dyskinesias	T2/FLAIR hyperintensity in periventricular region and basal ganglion	Antimeasles antibodies elevated	NA	Acute fulminant	Antiepileptics<br><br>intraventricular (IV) Interferon-a2B therapy	Akinetic mute
Malhotra and Garg 2015	India	25/M	Had measles at 3 years	3 months	Recurrent falls<br><br>Mental decline<br><br>Myoclonus	Slowness of movements<br><br>Body tilt	Pisa syndrome and striatal toe	T2/FLAIR hyperintensity in periventricular regions	Cells were elevated<br><br>Antimeasles antibodies elevated	NA	Acute fulminant	Clonazepam and Trihexyphenidyl<br><br>Interferon-alpha	Died

Kannan et al 2015	India	8/M	NA	1 month	Mental decline<br><br>Myoclonus	Abnormal twisting of lips and tongue with slurring of speech, drooling, and difficulty in swallowing<br><br>Bradykinesia, generalized dystonia, orofacial-dyskinesia, rigidity in all four limbs	Dystonia-Parkinsonism	Normal	Antimeasles antibodies elevated	NA	Acute fulminant	Antiepileptics	Further deterioration
Bozlu et al 2015	Turkey	12/M	Vaccinated	2 months	Periodic myoclonus	Difficulty in walking<br><br>Dystonia, left-sided<br><br>Tremors<br><br>Generalized stiffness	Juvenile Parkinson disease	T2/FLAIR hyperintensity in periventricular parietal regions	Antimeasles antibodies elevated	NA	Acute fulminant	Levodopa, trihexyphenidyl, tetrabenazine and clonazepam<br><br>IVIG	Improved
Serin et al 2014	Turkey	11/M	NA	1 week	Speech impairment and gait instability	Shaking in the right hand and limping in the right leg	Hemidystonia	T2/FLAIR hyperintensity in the basal ganglia and parieto-occipital region	Antimeasles antibodies elevated	NA	Chronic	Antiepileptics	Gradual deterioration
Roceanu et al 2013	Romania	19/M	Measles at 7 months	NA	Mental decline<br><br>Vision loss	Involuntary movements of the left upper limb<br><br>Unilateral myoclonic jerks choreoathet	Choreoathetosis and unilateral myoclonus	Normal	Antimeasles antibodies elevated	NA	NA	NA	NA

						osis of the left fingers							
Dey and Bhattacharya 2013	India	10/M	Had measles at 2 years	5 months	Ataxia Periodic myoclonus	Complex tics involving shoulder and facial muscles<br><br>Stereotypic repetitive movements and bradykinesia	Tics	T2/FLAIR hyperintensity in parieto-occipital region	Antimeasles antibodies elevated	NA	Chronic	Isoprinosine<br><br>Antiepileptics	NA
Yiş 2012	Turkey	14/M	NA	2 years	Drop attacks and behavioral changes<br><br>Rapidly progressive dystonia, hyperpyrexia	Paroxysmal dystonic attacks	Status Dystonicus<br><br>Myoglobinuria	NA	NA	NA	NA	Isoprinosine<br><br>Carbamazepine<br><br>Baclofen	NA
Almeida et al 2012	Brazil	15/M	vaccinated	3 years	Seizures Myoclonus Mental decline	Spontaneous laughter Tongue tremor Dystonia in upper and lower limbs	Generalised dystonia	T2/FLAIR hyperintensity in both basal ganglion	Elevated protein<br><br>Antimeasles antibodies elevated	Brain autopsy=perivascular inflammatory cuffing, astro-microgliosis, neurono-phagia and Cowdry type A eosinophilic intranuclear inclusion bodies<br><br>Immunohistochemistry= measles	Chronic	Symptomatic	Died
Teber et al	Turkey	11/M	Vaccinated	1 week	Involuntary movement	Chorea on upper	Chorea	Normal	Antimeasles	NA	Acute fulminant	Haloperidol	Died

2011			Had measles at 3 years		and gait disturbance<br><br>Complex partial seizure with secondary Generalization<br><br>One month later progressive diffuse encephalopathy	extremities and on his tongue			antibodies elevated			Isoprinosine<br><br>Carbamazepine<br><br>Clonazepam	
Fabian et al 2009	Australia	22/F	NA	6 months	Vision loss Tremors Mental decline	Choreiform movements of the left upper Limb<br><br>dystonia of the left leg	Chorea and focal dystonia	T2/FLAIR hyperintensity in occipital lobes	Normal<br><br>Antimeasles antibodies elevated	Brain autopsy= cellular infiltration astrogliosis, cortical neuronal loss, microglial nodules and perivascular cuffing by lymphocytes<br><br>Neuronal intranuclear eosinophilic inclusions were seen only within the hippocampus<br><br>Electron microscopy= viral nucleocapsids of a paramyxovirus.	Acute fulminant	Supportive	Died

Misra et al 2008	India	13/M	NA	2 months	Difficulty in walking<br><br>Speech abnormality<br><br>Bradykinesia	Mask-like facies, Tremor, cogwheel rigidity, bradykinesia, and postural instability	Parkinsonism	Normal	Antimeasles antibodies elevated	NA	Acute fulminant	Levodopa and carbidopa	NA
		15/M	Vaccinated	3 months	Recurrent falls<br><br>Difficulty in walking<br><br>Speech abnormality<br><br>Mental decline<br><br>Periodic myoclonus	Masked facies<br><br>Hypophonic speech<br><br>Cogwheel rigidity, bradykinesia and impaired postural reflexes	Parkinsonism	Normal	Antimeasles antibodies elevated	NA	Acute fulminant	NA	NA
Ondo and Verma 2002	USA	26/F	NA	4 months	Vision loss Recurrent falls<br><br>Progressive mental decline	Intermittent neck turning facial grimacing and then arm and leg extension and twisting<br><br>Dystonic extension and rotation of the arms, extension of the right leg, rotation of the neck, and contraction of face	Generalised dystonia	Normal	Antimeasles antibodies elevated	NA	Acute fulminant	Antiepileptics<br><br>Antiviral medication, amantadine, and prednisone	Died
Scheidt et al 2001	Germany	15/M	NA	1 year	Gait unsteadiness	Dystonia in his left hand	Focal dystonia	T2/FLAIR hyperintensity in the	Normal<br><br>Antimea	NA	Chronic	Isoprinosine	Akinetic mute

					Falls<br><br>Speech difficulties<br><br>Progressive mental decline<br><br>Myoclonus			thalamic and hypothalamic regions<br>Later in brain stem and periventricular regions	sles antibodies elevated				
Dimova and Bojinova 2000<br>A report of 3 patientS, 2 had movement disorders.	Turkey	11/F	Had measles at 2 years<br><br>Unvaccinated	Sudden	Progressive encephalopathy<br><br>Seizures<br>Periodic myoclonus	Action tremor in the right hand	Tremors and hyperkinesia	T2/FLAIR hyperintensity in whole of right frontal lobe	Antimeasles antibodies elevated	NA	Acute fulminant	Isoprinosine	Akinetic mute
		6/F	Had measles at 6 months<br><br>Unvaccinated	NA	Progressive encephalopathy<br><br>Periodic myoclonus	Left-leg dystonia and hemi parkinsonism.	Hemidystonia- hemi parkinsonism	T2/FLAIR hyperintensity in parieto-occipital region	Antimeasles antibodies elevated	NA	Chronic	Isoprinosine	NA
Vela et al 1997	Spain	30/M	Had measles at 8 months<br><br>Unvaccinated	3 years	Movements of neck that were considered as “tics”.<br><br>Periodic myoclonus<br><br>Encephalopathy	Movements of neck that were considered as “tics”.	Tics	T2/FLAIR hyperintensity in parieto-occipital region	Antimeasles antibodies elevated	NA	Chronic	Carbamazepine<br><br>Isoprinosine	Akinetic mute
Doh et al 1997	Korea	26/M	Had measles at 13 years	1 month	Bradykinesia<br>Slurred speech<br>Gait abnormality<br>Myoclonic jerks	Masked facies<br>Bradykinesia<br>Rigidity<br>Parkinsonian gait	Parkinsonism	Normal later brain atrophy	Antimeasles antibodies elevated	Brain biopsy- glial and inflammatory changes<br>Neuronal loss<br>Perivascular cuffing<br>Demyelination<br><br>Inclusions were not seen.	Chronic	Interferon<br>Amantadine<br>Isoprinosine<br>Rifampicin	Died
Jankovic 1988	USA	16/M<br>Delay	NA	2 years	Left sided weakness	Bradykinesia	Parkinsonism	T2/FLAIR hyperinten	Antimeasles	Brain biopsy- glial and	Chronic	Carbidopa-	Stabilised

		ed milestones			Gait abnormality<br><br>Chorioretinal scars	Cog wheel<br>Rigidity<br>Tremors<br>Masked facies<br>Sialorrhea<br>Stooped posture<br>Retropulsion		sity in parieto-occipital region	antibodies elevated	inflammatory changes<br>Neuronal loss<br>Perivascular cuffing<br>Demyelination<br><br>Inclusion bodies were seen.<br><br>Electron microscopy= viral nucleocapsids of a paramyxovirus		levodopa<br><br>Amantadine	

References

Youron P, Mahajan S, Balaini N, Mehta S, Lal V. Fulminant SSPE Presenting as a Hyperkinetic Movement Disorder. Mov Disord Clin Pract. 2023;10(5):830-832. doi: 10.1002/mdc3.13686.

Mondal R, Deb S, Mahata M, Saha S, Lahiri D, Benito-León J. Subacute Sclerosing Panencephalitis in a 63-Year-Old Woman Presenting as Generalized Choreoathetosis. Neurohospitalist. 2023 Oct;13(4):381-393. doi: 10.1177/19418744231177105.

Kaur S, Singh AS, Prabhakar S, Singhvi JP, Mann HS, Kaul A. Pisa Syndrome in Subacute Sclerosing Panencephalitis: A Case Report and Review of the Literature. J Mov Disord. 2023 Sep;16(3):336-338. doi: 10.14802/jmd.23052.

Harikrishna GV, Chowdary MR, Vengalil S, Nalini A, Yadav R. Status Dystonicus in Subacute Sclerosing Panencephalitis-A Rare Presentation in Emergency. Neurol India. 2023 Sep-Oct;71(5):994-997. doi: 10.4103/0028-3886.388104.

Garg RK, Pandey S, Nigam H, Keerthiraj DB, Rizvi I, Kumar N, Uniyal R, Malhotra HS, Sharma PK. Case Report: An Unusual Case of Subacute Sclerosing Panencephalitis with Distinctive Clinical and Neuroimaging Features. Am J Trop Med Hyg. 2023 Mar 13;108(5):1025-1027. doi: 10.4269/ajtmh.22-0731.

Garg D, Kakkar V, Sharma S. Periodic Laughter-like Episodes in Subacute Sclerosing Panencephalitis-Is it Gelastic Myoclonus? Mov Disord Clin Pract. 2023 Aug 7;10(10):1547-1548. doi: 10.1002/mdc3.13848.

Kalita J, Chaudhary SK, Kumar B, Jadhav M. Case Report: Focal Myoclonus with a Striatal Lesion as a Presentation of Subacute Sclerosing Panencephalitis. Am J Trop Med Hyg. 2022 May 9;106(6):1729–31. doi: 10.4269/ajtmh.22-0046.

Holla VV, Chaithra SP, Prasad S, Kamble N, Pal PK, Yadav R. Faciobrachial dystonic seizure-like events in a patient with subacute sclerosing panencephalitis. Annals of Movement Disorders. 2022 May 1;5(2):121-4. DOI: 10.4103/AOMD.AOMD_41_21

Cornelius LP, Elango N, Jeyaram VK. Akinetic rigid syndrome as a presenting feature of subacute sclerosing pan encephalitis. Neurology Asia. 2022 Mar 31;27(1):191-4.

Regmi J, Airani ZK, Mohan S, Patil S, Shetty V, Moulick N. Subacute Sclerosing Panencephalitis Presenting Like Parkinsonism: A Rare Case. International Journal of Science and Research. 2020; 10:1192-1193. DOI:10.21275/SR211225090518

Uniyal R, Garg RK, Malhotra HS, Tejan N, Kumar N, Pandey S, Shekhar R. A Case of Subacute Sclerosing Panencephalitis: Some Unusual Clinico-Radiological Manifestations. Neurol India. 2021 Sep-Oct;69(5):1446-1448. doi: 10.4103/0028-3886.329564.

Reddy RB, Joshi D, Kumar A. Subacute Sclerosing Panencephalitis with An Atypical Presentation. Ann Indian Acad Neurol. 2021 Nov-Dec;24(6):946-947. doi: 10.4103/aian.AIAN_791_20.

Khilari ML, Sharma PK. Clinical conundrum: status epilepticus culminating into acute dystonia myoclonus. BMJ Case Rep. 2020 Feb 28;13(2):e233397. doi: 10.1136/bcr-2019-233397.

Guruswamy A, Kurpad K P. Interesting MRI finding in SSPE – a case report. Polish Annals of Medicine. 2020;27(1):45-7. https://doi.org/10.29089/2019.19.00088

Tandra HV, Roy PS, Sharma R, Bhatia V, Saini AG. Subacute Sclerosing Panencephalitis Presenting as Choreoathetosis and Basal Ganglia Hyperintensities. Neurohospitalist. 2019 Jan;9(1):26-29. doi: 10.1177/1941874418776902.

Pandey S, Tomar LR, Tater P. Pisa Syndrome in a Child With Subacute Sclerosing Panencephalitis. JAMA Neurol. 2018 Feb 1;75(2):255-256. doi: 10.1001/jamaneurol.2017.4092.

Goswami JN, Roy S. Dystonic Storm: An Atypical Presentation of Subacute Sclerosing Panencephalitis. Indian Pediatr. 2018 May 15;55(5):441.

Garg D, Reddy V, Singh RK, Dash D, Bhatia R, Tripathi M. Neuroleptic malignant syndrome as a presenting feature of subacute sclerosing panencephalitis. J Neurovirol. 2018 Feb;24(1):128-131. doi: 10.1007/s13365-017-0602-4.

Singhi P, Saini AG, Sankhyan N, Gupta P, Vyas S. Blindness, dancing extremities, and corpus callosum and brain stem involvement: an unusual presentation of fulminant subacute sclerosing panencephalitis. J Child Neurol. 2015 Jan;30(1):87-90. doi: 10.1177/0883073813520498.

Raina GB, Folgar SS, Garrido JP, Calvo DS, Olivos NA, Morera N, Moreno M, Roca MU, Micheli F. Secondary kinesigenic paroxysmal dyskinesias: Report of two unusual cases responsive to carbamazepine. Basal Ganglia. 2015 Mar 1;5(1):7-9.

Malhotra HS, Garg RK. Pearls & Oy-sters: Pisa syndrome: an unusual feature of adult-onset fulminant SSPE. Neurology. 2015 Jan 20;84(3):e12-4. doi: 10.1212/WNL.0000000000001161.

Kannan L, Jain P, Sharma S, Gulati S. Subacute sclerosing panencephalitis masquerading as rapid-onset dystonia-Parkinsonism in a child. Neurol India. 2015 Jan-Feb;63(1):109-10. doi: 10.4103/0028-3886.152678.

Bozlu G, Cobanogullari Direk M, Okuyaz C. Subacute sclerosing panencephalitis with parkinsonian features in a child: A case report. Brain Dev. 2015 Oct;37(9):901-3. doi: 10.1016/j.braindev.2015.02.008.

Serin HM, Bilen S, Cansu A. Subacute Sclerosing Panencephalitis Presenting with Hemidystonia. Medical Bulletin of Haseki/Haseki Tip Bulteni. 2014 Jun 1;52(2). DOI: 10.4274/haseki.1451
Roceanu A, Antochi F, Bajenaru O. Atypical clinical presentation of subacute sclerosing panencephalitis (SSPE). Romanian Journal of Neurology/ Revista Romana de Neurologie 2013;12:142-147.

Dey PK, Bhattacharya T. Subacute sclerosing panencephalitis with tics as first symptom. Indian Pediatr. 2013 Nov 8;50(11):1067-8.

Yiş U. Status dystonicus and rhabdomyolysis in a patient with subacute sclerosing panencephalitis. Turk J Pediatr. 2012 Jan-Feb;54(1):90-1.

Almeida KJ, Brucki SMD, Duarte MIS, Pasqualucci CAG, Rosemberg S, Nitrini R. Basal ganglia lesions in subacute sclerosing panencephalitis. Dement Neuropsychol. 2012 Oct-Dec;6(4):286-289. doi: 10.1590/S1980-57642012DN06040014.

Teber S, Sezer T, Kafali M, Deda G. Subacute sclerosing panencephalitis with an atypical presentation: A case report. Journal of Pediatric Neurology 2011;9:127-130.

Fabian VA, Lee HY, Keith-Rokosh JL, de Souza JL, Stewart-Wynne E. A 22-year-old Australian woman with atypical subacute sclerosing panencephalitis diagnosed at postmortem. J Clin Neurosci 2010;17:1192-1194.

Misra AK, Roy A, Das SK. Parkinsonian presentation of SSPE: Report of two cases. Neurology Asia 2008;13:117-120.

Ondo WG, Verma A. Physiological assessment of paroxysmal dystonia secondary to subacute sclerosing panencephalitis. Mov Disord. 2002 Jan;17(1):154-7. doi: 10.1002/mds.10005.

Scheidt R, Schellenschmitt M, Dorstelmann D. Behavioural disturbances and dystonia as first manifestations of subacute sclerosing panencephalitis. AKTUELLE NEUROLOGIE. 2001 Mar 1;28(2):82-5.

Dimova P, Bojinova V. Subacute sclerosing panencephalitis with atypical onset: clinical, computed tomographic, and magnetic resonance imaging correlations. J Child Neurol. 2000 Apr;15(4):258-60. doi: 10.1177/088307380001500411.

Vela L, Garcia-Merino A, Escamilla C. Adult-onset subacute sclerosing panencephalitis first seen as craniocervical myoclonus. Mov Disord. 1997 May;12(3):462-4. doi: 10.1002/mds.870120335.

DOH W-B, KIM S-M, KIM S-Y, et al. Subacute sclerosing panencephalitis presenting as young adult onset parkinsonism. Journal of the Korean Neurological Association 1997:874-880.

Jankovic J, Armstrong D, Low NL, Goetz CG. Case 2, 1988. Congenital mental retardation and juvenile parkinsonism. Mov Disord. 1988;3(4):352-61. doi: 10.1002/mds.870030413.
