# Supplementary material for: Hyperkinetic and Hypokinetic Movement Disorders in SSPE: A Systematic Review of Case Reports and Case Series
Source: Tremor Other Hyperkinet Mov (N Y). 2024 May 14;14:27. doi: 10.5334/tohm.875 (PMC11100530; doi:10.5334/tohm.875)
Supplement: Supplementary File. — Supplementary Tables 1 to 4. [file tohm-14-1-875-s1.zip › tohm-875_garg_s1/Supplementary Table-4.docx]

**Supplementary Table-4: Hyperkinetic and Hypokinetic Movement Disorders in SSPE: A Systematic Review of Case Reports and Case Series**

**Quality assessment**

| **References** | **Number of cases** | **Does the patient represent the whole experience of the investigator** | **Was the exposure adequately ascertained?** | **Was the outcome adequately ascertained?** | **Were other alternative causes that may explain the observation ruled out?** | **Was there a challenge and/or re-challenge phenomenon?** | **Was there a dose-response effect?** | **Was follow-up long enough for outcomes to occur?** | **Is the case(s) described with sufficient details to allow practitioners make inferences related to their own practice?** | **Score** |
| --- | --- | --- | --- | --- | --- | --- | --- | --- | --- | --- |
| Youron et al 2023 | **1** | **Yes** | **Yes** | **No** | **Yes** | **No** | **No** | **Yes** | **Yes** | **5** |
| Mondal et al 2023 |  | **Yes** | **Yes** | **Yes** | **Yes** | **No** | **No** | **Yes** | **Yes** | **6** |
| Kaur et al 2023 |  | **Yes** | **Yes** | **Yes** | **Yes** | **No** | **No** | **Yes** | **Yes** | **6** |
| Harikrishna et al 2023 |  | **Yes** | **Yes** | **Yes** | **Yes** | **No** | **No** | **Yes** | **Yes** | **6** |
| Garg et al 2023 |  | **Yes** | **Yes** | **Yes** | **Yes** | **No** | **No** | **Yes** | **Yes** | **6** |
| Garg et al 2023 | **1** | **Yes** | **Yes** | **Yes** | **Yes** | **No** | **No** | **Yes** | **Yes** | **6** |
| Kalita et al 2022 | **1** | **Yes** | **Yes** | **Yes** | **Yes** | **No** | **No** | **Yes** | **Yes** | **6** |
| Holla et al 2022 | **1** | **Yes** | **Yes** | **Yes** | **Yes** | **No** | **No** | **Yes** | **Yes** | **6** |
| Cornelius et al 2022 | **1** | **Yes** | **Yes** | **Yes** | **Yes** | **No** | **No** | **Yes** | **Yes** | **6** |
| Regmi et al 2021 | **1** | **Yes** | **Yes** | **Yes** | **Yes** | **No** | **No** | **Yes** | **Yes** | **6** |
| Uniyal et al 2021 | **1** | **Yes** | **Yes** | **Yes** | **Yes** | **No** | **No** | **Yes** | **Yes** | **6** |
| Reddy et al 2021 | **1** | **Yes** | **Yes** | **No** | **Yes** | **No** | **No** | **Yes** | **Yes** | **5** |
| Khilari et al 2020 | **1** | **Yes** | **Yes** | **Yes** | **Yes** | **No** | **No** | **Yes** | **Yes** | **6** |
| Guruswamy and Kurpad 2020 | **1** | **Yes** | **Yes** | **No** | **Yes** | **No** | **No** | **Yes** | **Yes** | **5** |
| Tandra et al 2019 | **1** | **Yes** | **Yes** | **Yes** | **Yes** | **No** | **No** | **Yes** | **Yes** | **6** |
| Pandey et al 2018 | **1** | **Yes** | **Yes** | **Yes** | **Yes** | **No** | **No** | **Yes** | **Yes** | **6** |
| Goswami and Roy 2018 | **1** | **Yes** | **Yes** | **Yes** | **Yes** | **No** | **No** | **Yes** | **Yes** | **6** |
| Garg et al 2018 | **1** | **Yes** | **Yes** | **Yes** | **Yes** | **No** | **No** | **Yes** | **Yes** | **6** |
| Singhi et al 2015 | **1** | **Yes** | **Yes** | **Yes** | **Yes** | **No** | **No** | **Yes** | **Yes** | **6** |
| Raina et al 2015 | **1** | **Yes** | **Yes** | **Yes** | **Yes** | **No** | **No** | **Yes** | **Yes** | **6** |
| Malhotra and Garg 2015 | **1** | **Yes** | **Yes** | **Yes** | **Yes** | **No** | **No** | **Yes** | **Yes** | **6** |
| Kannan et al 2015 | **1** | **Yes** | **Yes** | **Yes** | **Yes** | **No** | **No** | **Yes** | **Yes** | **6** |
| Bozlu et al 2015 | **1** | **Yes** | **Yes** | **Yes** | **Yes** | **No** | **No** | **Yes** | **Yes** | **6** |
| Serin et al 2014 | **1** | **Yes** | **Yes** | **Yes** | **Yes** | **No** | **No** | **Yes** | **Yes** | **6** |
| Roceanu et al 2013 | **1** | **Yes** | **Yes** | **No** | **Yes** | **No** | **No** | **Yes** | **Yes** | **5** |
| Dey and Bhattacharya 2013 | **1** | **Yes** | **Yes** | **No** | **Yes** | **No** | **No** | **Yes** | **Yes** | **5** |
| Yiş 2012 | **1** | **Yes** | **Yes** | **No** | **Yes** | **No** | **No** | **Yes** | **Yes** | **5** |
| Almeida et al 2012 | **1** | **Yes** | **Yes** | **Yes** | **Yes** | **No** | **No** | **Yes** | **Yes** | **6** |
| Teber et al 2011 | **1** | **Yes** | **Yes** | **Yes** | **Yes** | **No** | **No** | **Yes** | **Yes** | **6** |
| Fabian et al 2009 | **1** | **Yes** | **Yes** | **Yes** | **Yes** | **No** | **No** | **Yes** | **Yes** | **6** |
| Misra et al 2008 | **1** | **Yes** | **Yes** | **No** | **Yes** | **No** | **No** | **Yes** | **Yes** | **5** |
|  | **2** | **Yes** | **Yes** | **No** | **Yes** | **No** | **No** | **Yes** | **Yes** | **5** |
| Ondo and Verma 2002 | **1** | **Yes** | **Yes** | **Yes** | **Yes** | **No** | **No** | **Yes** | **Yes** | **6** |
| Scheidt et al 2001 | **1** | **Yes** | **Yes** | **Yes** | **Yes** | **No** | **No** | **Yes** | **Yes** | **6** |
| Dimova and Bojinova 2000 | **1** | **Yes** | **Yes** | **Yes** | **Yes** | **No** | **No** | **Yes** | **Yes** | **6** |
|  | **2** | **Yes** | **Yes** | **No** | **Yes** | **No** | **No** | **Yes** | **Yes** | **5** |
| Vela et al 1997 | **1** | **Yes** | **Yes** | **Yes** | **Yes** | **No** | **No** | **Yes** | **Yes** | **6** |
| Doh et al 1997 | **1** | **Yes** | **Yes** | **Yes** | **Yes** | **No** | **No** | **Yes** | **Yes** | **6** |
| Jankovic 1988 | **1** | **Yes** | **Yes** | **Yes** | **Yes** | **No** | **No** | **Yes** | **Yes** | **6** |
