# Supplementary material for: Hyperkinetic and Hypokinetic Movement Disorders in SSPE: A Systematic Review of Case Reports and Case Series
Source: Tremor Other Hyperkinet Mov (N Y). 2024 May 14;14:27. doi: 10.5334/tohm.875 (PMC11100530; doi:10.5334/tohm.875)
Supplement: Supplementary File. — Supplementary Tables 1 to 4. [file tohm-14-1-875-s1.zip › tohm-875_garg_s1/Supplementary Table-4.pdf]

Supplementary Table-4: Hyperkinetic and Hypokinetic Movement Disorders in SSPE: A Systematic Review of Case Reports and Case Series

Quality assessment

References	Number of cases	Does the patient represent the whole experience of the investigator	Was the exposure adequately ascertained?	Was the outcome adequately ascertained?	Were other alternative causes that may explain the observation ruled out?	Was there a challenge and/or re-challenge phenomenon?	Was there a dose-response effect?	Was follow-up long enough for outcomes to occur?	Is the case(s) described with sufficient details to allow practitioners make inferences related to their own practice?	Score
Youron et al 2023	1	Yes	Yes	No	Yes	No	No	Yes	Yes	5
Mondal et al 2023		Yes	Yes	Yes	Yes	No	No	Yes	Yes	6
Kaur et al 2023		Yes	Yes	Yes	Yes	No	No	Yes	Yes	6
Harikrishna et al 2023		Yes	Yes	Yes	Yes	No	No	Yes	Yes	6
Garg et al 2023		Yes	Yes	Yes	Yes	No	No	Yes	Yes	6
Garg et al 2023	1	Yes	Yes	Yes	Yes	No	No	Yes	Yes	6
Kalita et al 2022	1	Yes	Yes	Yes	Yes	No	No	Yes	Yes	6
Holla et al 2022	1	Yes	Yes	Yes	Yes	No	No	Yes	Yes	6
Cornelius et al 2022	1	Yes	Yes	Yes	Yes	No	No	Yes	Yes	6
Regmi et al 2021	1	Yes	Yes	Yes	Yes	No	No	Yes	Yes	6
Uniyal et al 2021	1	Yes	Yes	Yes	Yes	No	No	Yes	Yes	6
Reddy et al 2021	1	Yes	Yes	No	Yes	No	No	Yes	Yes	5

Khilari et al 2020	**1**	**Yes**	**Yes**	**Yes**	**Yes**	**No**	**No**	**Yes**	**Yes**	**6**
Guruswamy and Kurpad 2020	**1**	**Yes**	**Yes**	**No**	**Yes**	**No**	**No**	**Yes**	**Yes**	**5**
Tandra et al 2019	**1**	**Yes**	**Yes**	**Yes**	**Yes**	**No**	**No**	**Yes**	**Yes**	**6**
Pandey et al 2018	**1**	**Yes**	**Yes**	**Yes**	**Yes**	**No**	**No**	**Yes**	**Yes**	**6**
Goswami and Roy 2018	**1**	**Yes**	**Yes**	**Yes**	**Yes**	**No**	**No**	**Yes**	**Yes**	**6**
Garg et al 2018	**1**	**Yes**	**Yes**	**Yes**	**Yes**	**No**	**No**	**Yes**	**Yes**	**6**
Singhi et al 2015	**1**	**Yes**	**Yes**	**Yes**	**Yes**	**No**	**No**	**Yes**	**Yes**	**6**
Raina et al 2015	**1**	**Yes**	**Yes**	**Yes**	**Yes**	**No**	**No**	**Yes**	**Yes**	**6**
Malhotra and Garg 2015	**1**	**Yes**	**Yes**	**Yes**	**Yes**	**No**	**No**	**Yes**	**Yes**	**6**
Kannan et al 2015	**1**	**Yes**	**Yes**	**Yes**	**Yes**	**No**	**No**	**Yes**	**Yes**	**6**
Bozlu et al 2015	**1**	**Yes**	**Yes**	**Yes**	**Yes**	**No**	**No**	**Yes**	**Yes**	**6**
Serin et al 2014	**1**	**Yes**	**Yes**	**Yes**	**Yes**	**No**	**No**	**Yes**	**Yes**	**6**
Roceanu et al 2013	**1**	**Yes**	**Yes**	**No**	**Yes**	**No**	**No**	**Yes**	**Yes**	**5**
Dey and Bhattacharya 2013	**1**	**Yes**	**Yes**	**No**	**Yes**	**No**	**No**	**Yes**	**Yes**	**5**
Yiş 2012	**1**	**Yes**	**Yes**	**No**	**Yes**	**No**	**No**	**Yes**	**Yes**	**5**
Almeida et al 2012	**1**	**Yes**	**Yes**	**Yes**	**Yes**	**No**	**No**	**Yes**	**Yes**	**6**
Teber et al 2011	**1**	**Yes**	**Yes**	**Yes**	**Yes**	**No**	**No**	**Yes**	**Yes**	**6**
Fabian et al 2009	**1**	**Yes**	**Yes**	**Yes**	**Yes**	**No**	**No**	**Yes**	**Yes**	**6**
Misra et al 2008	**1**	**Yes**	**Yes**	**No**	**Yes**	**No**	**No**	**Yes**	**Yes**	**5**
	**2**	**Yes**	**Yes**	**No**	**Yes**	**No**	**No**	**Yes**	**Yes**	**5**
Ondo and Verma 2002	**1**	**Yes**	**Yes**	**Yes**	**Yes**	**No**	**No**	**Yes**	**Yes**	**6**
Scheidt et al 2001	**1**	**Yes**	**Yes**	**Yes**	**Yes**	**No**	**No**	**Yes**	**Yes**	**6**

Dimova and Bojinova 2000	**1**	**Yes**	**Yes**	**Yes**	**Yes**	**No**	**No**	**Yes**	**Yes**	**6**
	**2**	**Yes**	**Yes**	**No**	**Yes**	**No**	**No**	**Yes**	**Yes**	**5**
Vela et al 1997	**1**	**Yes**	**Yes**	**Yes**	**Yes**	**No**	**No**	**Yes**	**Yes**	**6**
Doh et al 1997	**1**	**Yes**	**Yes**	**Yes**	**Yes**	**No**	**No**	**Yes**	**Yes**	**6**
Jankovic 1988	**1**	**Yes**	**Yes**	**Yes**	**Yes**	**No**	**No**	**Yes**	**Yes**	**6**
